# Color Intensity of the Red-Fleshed Berry Phenotype of *Vitis vinifera* Teinturier Grapes Varies Due to a 408 bp Duplication in the Promoter of *VvmybA1*

**DOI:** 10.3390/genes11080891

**Published:** 2020-08-05

**Authors:** Franco Röckel, Carina Moock, Ulrike Braun, Florian Schwander, Peter Cousins, Erika Maul, Reinhard Töpfer, Ludger Hausmann

**Affiliations:** 1 Institute for Grapevine Breeding Geilweilerhof, Julius Kühn-Institut, Federal Research Centre for Cultivated Plants, 76833 Siebeldingen, Germany; franco.roeckel@julius-kuehn.de (F.R.); carina.moock@julius-kuehn.de (C.M.); ulrike.braun@julius-kuehn.de (U.B.); florian.schwander@julius-kuehn.de (F.S.); erika.maul@julius-kuehn.de (E.M.); reinhard.toepfer@julius-kuehn.de (R.T.); 2 E. and J. Gallo Winery, Modesto, CA 95354, USA; peter.cousins@ejgallo.com

**Keywords:** anthocyanin, *Myb*, repetitive DNA element, periclinal chimera, genetic diversity, grapevine, teinturier, berry color, *Vitis*

## Abstract

Grapevine (*Vitis vinifera*) teinturier cultivars are characterized by their typical reddish leaves and red-fleshed berries due to ectopic anthocyanin formation. Wines of these varieties have economic importance as they can be used for blending to enhance the color of red wines. The unique and heritable mutation has been known for a long time but the underlying genetic mechanism still is not yet understood. Here we describe the association of the red-fleshed berry phenotype with a 408 bp repetitive DNA element in the promoter of the *VvmybA1* gene (grapevine color enhancer, GCE). Three different clones of ‘Teinturier’ were discovered with two, three and five allelic GCE repeats (*MybA1t2*, *MybA1t3* and *MybA1t5*). All three clones are periclinal chimeras; these clones share the same L1 layer, but have distinct L2 layers with different quantities of GCE repeats. Quantitative real time PCR and HPLC analysis of leaf and berry samples showed that the GCE repeat number strongly correlates with an increase of the expression of *VvmybA1* itself and the *VvUFGT* gene regulated by it and the anthocyanin content. A model is proposed based on autoregulation of *VvmybA1t* to explain the red phenotype which is similar to that of red-fleshed apples. This study presents results about the generation and modes of action of three *MybA1t* alleles responsible for the red-fleshed berry phenotype of teinturier grapevines.

## 1. Introduction

Anthocyanins (from Greek “anthos” = flower and “kyanos” = blue) represent a ubiquitous class of metabolites in nature [1]. They belong to the very large group of flavonoids, which includes more than 4000 known compounds that have been isolated from plants [2] and are among the most important pigments responsible for the coloration of plant tissues, especially fruits, flowers and leaves [3]. For grapevines (*Vitis vinifera* L.), the important fruit traits with a broad spectrum ranging from green/yellow to blue/black are linked with anthocyanin-based berry color formation [4]. The theoretically possible number of anthocyanins is about 100, consisting mainly of five basic anthocyanins (delphinidin-3-*O*-glucoside, cyanidin-3-*O*-glucoside, petunidin-3-*O*-glucoside, peonidin-3-*O*-glucoside and malvidin-3-*O*-glucoside) and their acylated derivatives [5,6].

The anthocyanin biosynthesis pathway shares with all flavonoids the same upstream pathway that branches at the level of flavanones [7,8]. The two key enzymes flavonoid 3’-hydroxylase (F3’H) and flavonoid 3’,5’-hydroxylase (F3′5′H) use the flavanone naringenin as the main substrate for a hydroxylation reaction. The resulting amounts of 3′-hydroxylated flavonoids and 3′,5′-hydroxylated flavonoids correlate with the expression level of the *F3′H* and *F3′5′H*, respectively [9,10]. All precursors of the anthocyanins are colorless up to a final glycosylation by UFGT (UDP-glucose: flavonoid-3-*O*-glucosyltransferase) resulting in the color-intensive pigments [11]. Further modification can occur by *O*-methyltransferases, which methylate hydroxyl groups at the 3′ and 5′ positions of the B ring [12] and acyltransferases, which attach acyl moieties (e.g., acetyl, coumaroyl or caffeoyl groups) to the C6′′ position of the glucose molecule [13]. Due to an increased intramolecular stacking, the acylation of anthocyanins is considered to contribute to enhanced stability and water solubility [14].

In grapevine, the relative abundance of anthocyanins and total quantities mainly are controlled genetically, developmentally regulated and variety-specific, but environmental factors such as light, temperature and water availability also influence the phenotype. Excessively high temperatures and too much light can have negative effects on berry color, whereas water deficit promotes berry anthocyanin content [15,16,17]. Additionally, intrinsic factors such as the concentration of the plant hormone abscisic acid (ABA) are linked to the levels of anthocyanins through upregulation of the expression of the key gene *VvUFGT* [18]. On genetic level, the expression of *VvUFGT* is controlled by a single locus on chromosome 2, also called berry color locus, at the physical position of about 14.2 Mb according to the reference genome sequence [19,20]. Furthermore, there are strong indications that this locus is a major genetic determinant of the ratio of 3′-hydroxylated/3′,5′-hydroxylated anthocyanins through regulation of *F3′H* and *F3′5′H* expression [10]. The color locus contains several *VvmybA* transcription factor genes of the R2R3-type with the key genes *VvmybA1* and *VvmybA2* active during ripening in the berry skin [21,22]. In contrast to colored grapes with two functional *VvmybA* genes, white varieties show only non-functional *VvmybA* genes at the berry color locus. In case of the non-functional *VvmybA1* allele, called *VvmybA1a* or *MybA1a*, the retrotransposon *Gret1* upstream of the coding sequence hampers the expression, whereas the functionality of *VvmybA2* is inactivated by two non-conservative mutations, an amino acid substitution and a frame shift, resulting in an early stop codon and a truncated protein. The functional *VvmybA1* gene instead, called *VvmybA1c* or *MybA1c*, completely lacks *Gret1* and is considered dominant over *VvmybA1a* [23].

**Figure 1:**
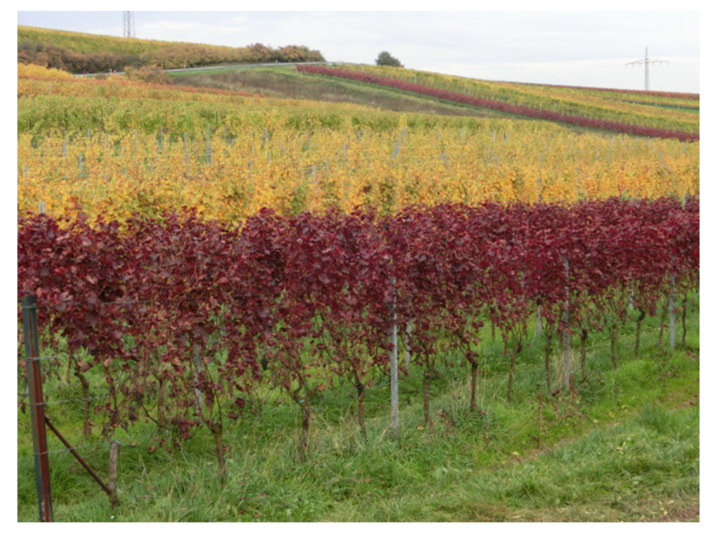
Typical dark red foliage coloration of a teinturier variety in early autumn (foreground and background), which is particularly striking in a mixed vineyard landscape.

Anthocyanins are synthesized typically in the skin but not in the pulp of grapevine berries starting with the onset of ripening (veraison = softening of the berry). In contrast, the grapevine variety ‘Teinturier (du Cher)’ and its derivatives (=teinturier varieties, also called dyers) deviate from this natural situation by accumulating variable anthocyanin contents in the leaves, the berry flesh and the berry skin. These varieties show in general a more or less intense red coloration of the foliage and stems during the growing season and dark red colored autumn leaves (Figure 1). The skin and pulp anthocyanin content differs with a higher total amount and higher proportion of dihydroxylated anthocyanins in the skin [24,25]. The first mention of ‘Teinturier’ alias ‘Negrier’ dates back to the 16th century, when Charles Estienne and Jean Liébault described the varieties grown in Burgundy (France) and stated that “only few plants are sufficient to blacken the other and to strengthen it” [26]. Since then, teinturier varieties have been used for blends to increase the color of pale red wines and remain economically important worldwide [27,28]. For example, ‘Rubired’, a teinturier variety descended from Teinturier’ that is chiefly cultivated in California (United States) represented 6.1% of the 2019 grape crush in that state, a greater fraction than other well-known internationally grown black grapes such as ‘Merlot’ and ‘Grenache’ respectively [29].

Berry color mutations are easily detectible in the vineyard by simple visual inspection; many clones with different colors than their parent variety have been selected since the rise of viticulture [30]. In this study, red fleshed clones and varieties descended from ‘Teinturier’ were the chief focus. Red fleshed varieties or clones have been identified from other lineages, not related to ‘Teinturier’, such as sports or clones from the varieties ‘Gamay Noir’ or ‘Summer Black’, but they show no direct relationship to the original teinturier varieties analyzed in this study [31,32]. Periclinal chimera sports in which the L1 layer is black fruited and the L2 layer is white fruited have been identified from several varieties, such as ‘Pinot Gris’ or ‘Malian’ (bud sports of ‘Pinot Noir’ and ‘Cabernet Sauvignon’, respectively) [33,34]. In these cases, a mutation occurred in the inner L2 layer of the apical meristem resulting in the loss of the functional berry color locus on chromosome 2. Since grapevine is propagated vegetatively, this kind of mutation can be stably maintained. Anthocyanins are only produced in the relatively thin L1 cell layer and the overall berry color appears to be grey [34]. Further alterations can follow based on cellular rearrangements between the two cell layers, either a displacement of the L1 cells leading to white berries or a replacement of the mutated L2 cells reverting again to dark colored berries [35].

The main goal of this study was to characterize the specific mutation of the grapevine teinturier varieties of the ‘Teinturier’ lineages differing in color intensity of their red-fleshed phenotype. Due to the altered organ-specific expression of *VvmybA1* we analyzed the coding sequence and the adjacent promoter region of a large set of teinturier varieties showing a range of berry and vine phenotypes. We compared red fleshed clones and varieties of the ‘Teinturier’ lineage to wild type varieties by examining the sequences of the *VvmybA1* region, expression data of anthocyanin biosynthesis genes, and anthocyanin content and composition of berry and leaf tissues. Based on these results, we propose a model that links the unique mutation on a genetic level with the distinct red berry flesh and other phenotypes of teinturier varieties.

## 2. Materials and Methods 

### 2.1. Plant Material and SSR Marker Analysis

‘Teinturier 3x’ and ‘Teinturier 5x’ (‘Rubintraube’) were obtained as dormant wood cuttings from the Bundessortenamt, Prüfstelle Hassloch, Germany and from the Staatliche Lehr- und Versuchsanstalt für Wein- und Obstbau, Weinsberg, Germany, respectively. All other grapevine accessions used were maintained in the vineyards of the germplasm repository at JKI Geilweilerhof, Siebeldingen, Germany. Potted plants were produced after leaf fall from woody cuttings during the winter season of 2015 and cultivated in the greenhouse. Berry samples were taken from field-grown vines; ten ripe berries of three sun-exposed bunches were collected at three developmental stages (four weeks before and after veraison, and at fully ripe stage (16–21° Brix)). Berry skin and flesh were immediately separated and the seeds were removed. Four to six leaves were randomly collected from six greenhouse-grown vines that were eight months old.

All samples for anthocyanin analysis were immediately weighed, freeze-dried and stored in the dark. The samples for transcript analysis were frozen in liquid nitrogen and stored at −80 °C. A complete material list with the types of tissues analyzed is shown in Table S1. The plant material was genotyped with nine genome-wide SSR (simple sequence repeat) markers to check the identity for trueness-to-type ([36], http://www.vivc.de/). Six additional SSR markers were used to determine the haplotype structure around the berry color locus on chromosome 2 (Table S2). The marker genotyping was carried out as described previously in Huber et al. [37].

### 2.2. DNA Preparation, PCR Analysis and Sequencing of VvmybA1 Alleles

Genomic DNA was isolated for a cell layer-specific PCR analysis as described in Vezzulli et al. [33]. We extracted DNA either from young leaves (L1 and L2 cell layers) or adventitious roots (L2 cell layer) of potted plants. We used the Plant DNA Mini Kit (Peqlab, Erlangen, Germany) following the supplier’s instructions. For amplification of the white Vvm*ybA1a* allele (VIT_02s0033g00410) the primers “a” and “c” were taken from Kobayashi et al. [38]. To ensure unique specificity for the amplification of the *VvmybA1c* allele, primers were located outside of the open reading frame. MybA1_prom_fw (5′-TCAGTGAGGGTAACAAAGTC-3′) and MybA1_prom_seq_fw (5′-TAAAGCCTATAATATTCCCAC-3′) were positioned in the 5′ flanking region 808 bp and 1237 bp upstream of the start codon and used for repeat number validation and sequencing, respectively. MybA1_term_rev (5′-CAAGCTAGAAAGAGAGATGTGT-3′) was used for both purposes and positioned in the 3′ flanking region 494 bp downstream of the stop codon. All primers were purchased from Metabion (Planegg, Germany). PCR for the repeat number validation was performed in a final reaction volume of 25 µL containing 20–30 ng genomic DNA, 0.3 mM dNTPs, 1 x KAPA HiFi Buffer, 0.3 mM of each primer and 0.5 U polymerase (KAPAHiFi^TM^ Hot-Start PCR-Kit from Peqlab, Erlangen, Germany). A Mastercycler^®^ gradient (Eppendorf AG, Hamburg, Germany) was used for amplification with the following touchdown cycle program: initial activation at 95 °C for 5 min; followed by 10 cycles of 98 °C for 20 s, 65 °C for 15 s (−0.5 °C each cycle temperature increment) and 72 °C for 120 s; followed by 20 cycles of 98 °C for 20 s, 60 °C for 15 s and 72 °C for 120 s; then a final extension at 72 °C for 5 min. To better determine the size of the amplified 5′ flanking regions of *VvmybA1* from ‘Teinturier’ clones, PCR products were purified with the Kit NucleoSpin^®^ Gel and PCR Clean-up (Macherey-Nagel, Düren, Germany) and restriction digested with *Eco*RI (Thermo Fisher Scientific, Darmstadt, Germany). DNA fragments were separated by electrophoresis on a 1% agarose gel (Biozym Scientific GmbH, Hessisch Oldendorf, Germany). For sequencing of the *VvmybA1* alleles, a Touchdown-PCR was conducted with the conditions described above. In the cycle program, the annealing temperature was 61 °C and the elongation time was increased to 150 s due to the longer PCR product. PCR products were purified and Sanger sequenced by GATC Biotech AG (Konstanz, Germany).

### 2.3. Extraction and Analysis of Anthocyanins

Anthocyanins were extracted from ground tissues (0.08–0.15 g) in 10 mL of an ethanol:water:hydrochloride acid solution (70:29:1) via overnight shaking at 100 rpm in the dark as previously detailed [39]. Extracts were centrifuged for 10 min at 11,000× *g* rpm and 50 µL of the supernatants were analyzed for anthocyanins on an Agilent 1100/1200 series HPLC system with a diode-array detector (Agilent Technologies, Palo Alto, Santa Clara, CA, USA). Separation was conducted on a LiChrospher 100 RP 18 (5 µm) column in LiChroCart 250-4 protected by a LiChroCart 4 mm RP 18 guard column (Merck, Darmstadt, Germany) as described in the OIV-MA-AS315-11 method [40]. A gradient consisting of solvent A (water/formic acid/acetonitrile, 87:10:3, *v*/*v*/*v*) and solvent B (water/formic acid/acetonitrile, 40:10:50, *v*/*v*/*v*) was applied at a flow rate of 0.4 mL/min and a isothermal column temperature of 20 °C as follows: 94–70% A and 6–30% B from 0 to 15 min, 70–50% A and 30–50% B from 15 to 30 min, 50–40% A and 50–60% B from 30 to 35 min, 40–94% A and 60–6% B from 35 to 41 min and a final step with 94% A and 6% B for re-equilibration of the column from 41 to 50 min. The detection wavelength for anthocyanins was set at 520 nm and the quantification was carried out by peak area measurements. The anthocyanin amount was expressed by using malvidin-3-O-glucoside as standard (Extrasynthese, Lyon, France) for a calibration curve (R^2^ = 0.99) as described in [41]. Identification and confirmation of anthocyanins was performed as previously described [42,43]. 

### 2.4. RNA Isolation, cDNA Synthesis and Quantitative Real-Time PCR (qRT-PCR)

Total RNA was extracted according to the protocol described by [44] with two changes: (1) the concentration of Tris-HCl was raised from 100 to 300 mM to increase the buffer capacity and keep the pH stable while suspending highly acidic tissues such as immature berries; (2) since the amount of starting material was scaled down from 3 g to 200 mg, the filtering step was omitted after lysis. The extracted RNA was treated with DNA-free DNase (Qiagen, Hilden, Germany) to remove any potential contamination by genomic DNA and afterwards purified with RNeasy^®^ MinElute^®^ Cleanup Kit (Qiagen) following the supplier’s protocol. For cDNA synthesis, 100–1000 ng of total RNA was reverse transcribed using the High Capacity cDNA Reverse Transcription Kit (Applied Biosystems, Carlsbad, CA, USA) following the manufacturer’s instructions. Quantitative real-time PCR for expression analysis was performed on an ABI 7500 Fast PCR System (Applied Biosystems, Darmstadt, Germany) using SYBR^®^-Green Real Time PCR Master Mix (Applied Biosystems, Darmstadt, Germany) with 10 ng cDNA and 250 nM primer in each reaction with the following thermocycling conditions: initial activation at 95 °C for 10 min; followed by 40 cycles of 95 °C for 15 s and 60 °C for 60 s; then a melting curve analysis according to the manufacturer’s instructions. Relative quantification was performed by means of the 2^−ΔΔCt^ method [45] using SDS software v1.4 (Applied Biosystems, Darmstadt, Germany). Expression data were normalized via geometric averaging to the expression of actin and GAPDH (glyceraldehyde 3-phosphate dehydrogenase) previously tested and recommended for gene expression analysis in grapevine berry development [46]. Primer sequences for actin were taken from [47], for GAPDH GAPDH_RT_fw (5′-TCAAGGTCAAGGACTCTAACACC-3′) and GAPDH_RT_rev (5′-CCAACAACGAACATAGGAGCA-3′). The primer pair for the expression analysis of *VvmybA1* was MybA_RT_fw (5′-GAGTTTGCATTAGACGAGGTT-3′) and MybA1_RT_rev (5′-CTTTTTGAAGTGGTGACT-3′). For *VvF3′H* and *VvF3′5′H* primer sequences were derived from [9], primers for *VvUFGT* were taken from [48].

### 2.5. Sequence Analysis and Statistics

Oligonucleotides were designed using CLC Main Workbench 7 (QIAGEN Bioinformatics, Aarhus, Denmark) based on the PN40024 grapevine reference genome [49,50] provided by Gramene (http://ensembl.gramene.org/Vitis_vinifera/Info/Index). The same software was used to assemble the sequence reads of the different *VvmybA1* alleles. The promoter sequence of the *VvmybA1* allele was screened for putative MYB protein binding domains with the databases PlantCARE [51] and PLACE [52]. For estimating the kinship of ‘Savagnin Blanc’, ‘Pinot Noir’” and ‘Teinturier’, ML-Relate software was used [53]. The SSR-marker dataset of [54] consisting of 1130 *V. vinifera* varieties, each genotyped with 20 SSR markers, was used to estimate allele frequencies within the *V. vinifera* population for a likelihood ratio test. The Student’s *t*-test and Tukey’s HSD test of the R-software environment were used to determine the statistical significant differences of anthocyanin and gene expression data [55].

## 3. Results

### 3.1. ‘Teinturier’ is a Periclinal Chimera and Related to ‘Pinot Noir’ and ‘Savagnin Blanc’

As reported in Jeong et al. [56] the red-fleshed phenotype of teinturier grapes may be caused by a mutation related to the organ-specific expression of *VvmybA1* at the color locus. For a detailed study of the molecular basis of this mutation, a set of 21 different grapevine accessions were chosen consisting of 15 ‘Teinturier’ clones, five varieties descended from ‘Teinturier’ through crossing or hybridization and ‘Pinot Noir’ as negative control. To verify the relatedness among the accessions, they were genotyped with nine genome-wide SSR markers and with six markers covering a region of about 327 kb around the berry color locus on chromosome 2 (Table S3). All ‘Teinturier’ accessions displayed the same marker allele pattern and the other varieties shared at least one allele, confirming the overall genetic identity of the ‘Teinturier’ clones and the close relationship between the teinturier varieties including ‘Pinot Noir’. At the chromosomal region around the berry color locus all 21 samples proved to be identical at the closest five of six marker sites. Since ‘Pinot Noir’ and ‘Teinturier’ are in a parent–offspring relationship to ‘Savagnin Blanc’ [54], the direct relationship was calculated with a likelihood ratio test showing that both varieties are most likely full-siblings with the parents ‘Savagnin Blanc’ x unknown (Table S4). 

**Figure 2:**
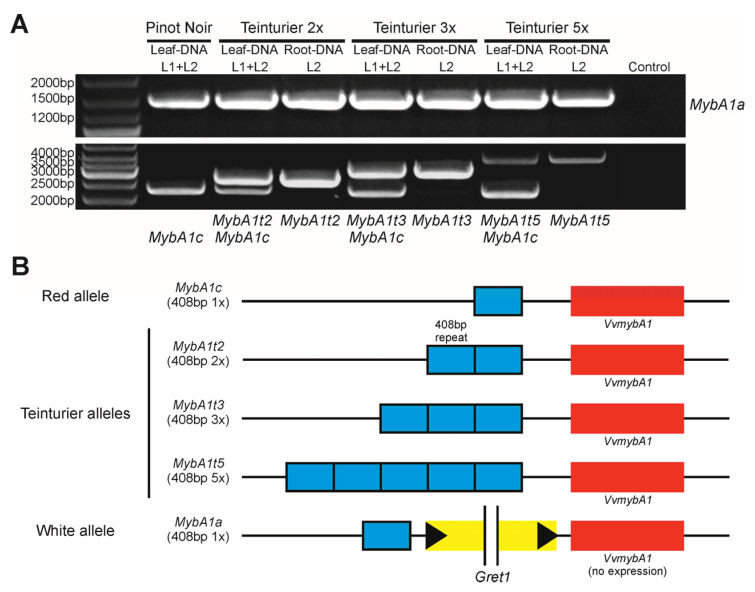
The tandem repeat mutation in the promoter region of *VvmybA1* specific for teinturier varieties. (**A**) PCR result of the white (*MybA1a*), red (*MybA1c*) and teinturier alleles (*MybA1t2*, *MybA1t3* and *MybA1t5*) of DNA prepared from leaves (L1 + L2) and roots (L2) of three different ‘Teinturier’ clones. ‘Pinot Noir’ as reference. (**B**) Schematic diagram of the three teinturier alleles found (*MybA1t2*, *MybA1t3* and *MybA1t5*) named based on the repeat number of the 408 bp GCE element (grapevine color enhancer) in the promoter region of *VvmybA1.* Red and white alleles as reference.

For the direct examination of the *VvmybA1* gene, the ‘Teinturier’ accessions were screened with PCR for their *MybA1* alleles. In contrast to the non-functional white allele *MybA1a*, primers for the functional *MybA1c* allele were designed to amplify the complete open reading frame and a larger part of the adjacent promoter. In all ‘Teinturier’ clones, three different *VvmybA1* amplicons were detected with a 1.55 kb and 2.2 kb PCR product shared by all samples (Figure 2A). The 1.55 kb PCR product corresponded to the 1559 bp white *MybA1a* allele [38] and the 2.2 kb PCR band was in accordance to the 2261 bp colored *MybA1c* allele [57]. Interestingly, a third unknown but specific *VvmybA1* PCR product was visible in the agarose gel with three distinct types of length polymorphisms (2.7, 3.1 and 3.9 kb). The observation of putative three alleles in genomic DNA of leaf tissue DNA consisting of L1 and L2 cell layers in ‘Teinturier’ resembled previous studies on grapevine cultivars ‘Pinot Meunier’ and ‘Pinot Gris’, proven to be periclinal chimeras [35,58].

In order to check whether the ‘Teinturier’ clones bear two genetically different cell layers, L2-specific root tissue DNA was compared to DNA from leaves (L1 + L2). The PCR analysis showed that in the L2-specific DNA samples the *MybA1c*-related 2.2 kb band was missing in all ‘Teinturier’ clones, whereas only the white *MybA1a* allele and one unknown allele variant could be amplified (Figure 2A). These results confirmed that the ‘Teinturier’ clones analyzed are periclinal chimeras bearing two genetically distinct cell layers: L1 carries *MybA1a* and *MybA1c*, most likely the original allele constitution, and L2 harbors *MybA1a* and a yet unknown new *MybA1* allele variant.

### 3.2. A Multiple Duplication of a 408 bp Promoter Fragment Is Associated with the Red-Fleshed Berry Phenotype 

The three new putative *VvmybA1* allele variants of the ‘Teinturier’ clones were sequenced to verify their identity and to search for the reason for the length polymorphisms. Sequence comparison of these new allele variants with the *VvmybA1c* allele of ‘Pinot Noir’ and that of the L1 layer of ‘Teinturier’ showed perfect identity, thereby supporting the full-sibling kinship of ‘Pinot Noir’ and ‘Teinturier’ as mentioned before. An alignment of the *MybA1c* allele of ‘Pinot Noir’ and the unknown allele sequence of ‘Teinturier’ is shown in Figure S1. However, a 408 bp insertion was found 338 bp upstream of the start codon in the promoter region of the smallest new allele. This segment was identified as a perfect directly orientated tandem repeat of the sequence 338–746 bp upstream of the ATG start codon. The sequences of the other two new alleles differed only in the number of repeats and revealed three and five copies, respectively (Figure 2B). Based on the Sanger sequencing, it was initially not unequivocally possible to determine the exact repeat number of the largest new allele variant due to the repetitive structure. Therefore, all PCR products were restriction digested with *Eco*RI and separated on an agarose gel for better estimating the size of fragments. The unique *Eco*RI site was located 22 bp downstream of the repeat site and cleaved the PCR products in a constant 3′ fragment (1768 bp) and a variable 5′ fragment of 493 bp (wildtype), 901 bp (two repeats), 1309 bp (three repeats) and 2125 bp (five repeats) (Figure S2). The three new alleles of the ‘Teinturier’ clones were named *MybA1t2*, *MybA1t3* and *MybA1t5* (*t* = teinturier) indicating the origin and the number of repeats.

Ten of the analyzed ‘Teinturier’ accessions possessed the *MybA1t2* allele, three *MybA1t3* and two *MybA1t5* (Table 1). The specific *MybA1t* alleles also were consistently identified in the 24 descendants of ‘Teinturier’ with red-fleshed berry phenotypes that were analyzed, but not in typical black fruited grapevine cultivars, such as ‘Pinot Noir’, or non-related red-fleshed accessions, such as ‘Pinot Teinturier’” or ‘Gamay Teinturier de Bouze’. These results clearly indicate that the repeated sequence element (named GCE for grapevine color enhancer) in the 5′ upstream region of the *VvmybA1t* alleles is associated with the colored berry flesh phenotype in teinturier varieties.

### 3.3. The Anthocyanin Concentration in Leaves Is Related to the GCE Number

**Figure 3:**
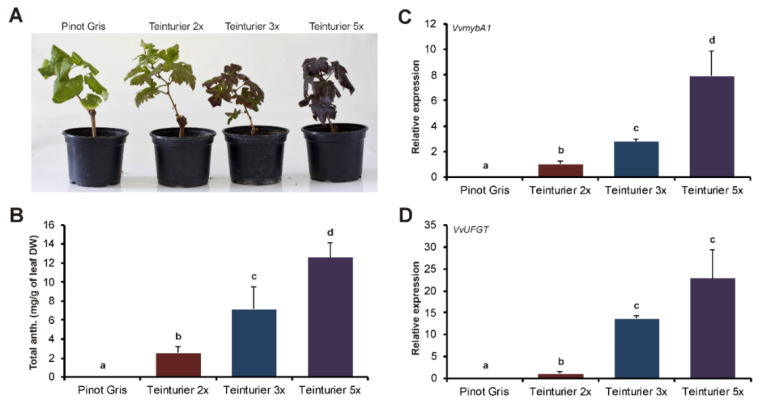
Total anthocyanin content and expression of *VvmybA1* and *VvUFGT* in leaf samples of greenhouse grown ‘Teinturier’ clones with increasing GCE number. ‘Pinot Gris’ as reference. (**A**) Greenhouse grown ‘Teinturier’ clones with colored leaves in comparison to ‘Pinot Gris’. (**B**) Total anthocyanin content of leaves. Data represent mean values of six independent replicates; error bars represent the standard deviation. (**C**,**D**) Relative expression of *VvmybA1* and *VvUFGT* normalized to the expression of ‘Teinturier 2x’. Data represent mean values of six independent replicates; error bars represent standard errors. Different letters indicate statistically significant differences with a *p*-value < 0.05 (Student’s *t*-test, Tukey’s HSD test).

In earlier observations, the two Teinturier’ clones ‘Teinturier femelle’ and ‘Teinturier mâle’ were described as differing only in the anthocyanin content of the whole plant, with the latter one being more intense colored [27]. The presence of two repeats in the first and three repeats in the *VvmybA1t* promoter of the latter accession suggested that the number of repeats affects the anthocyanin content. To test this hypothesis, the near-isogenic ‘Teinturier’ clones differing only in their GCE number (2x = *MybA1t2*, 3x = *MybA1t3* and 5x = *MybA1t5*) were cultivated in the greenhouse. The visual inspection of the eight-month-old plants showed enhanced leaf coloration with increased GCE number (Figure 3A). Subsequent HPLC analysis of the concentration of accumulated anthocyanins in leaves confirmed this first impression. The average leaf anthocyanin content increased significantly from the negative control ‘Pinot Gris’ to the ‘Teinturier’ clones (Figure 3B). From ‘Teinturier 2x’ to ‘Teinturier 3x’, the average leaf anthocyanin content increased by a factor of about 2.8 and from ‘Teinturier 3x’ to ‘Teinturier 5x’ of about 1.8. The same samples were used to determine the relative expression rate of *VvmybA1* and its downstream target *VvUFGT* using qRT-PCR. Expression of both genes reflected the differences in leaf anthocyanin content among the accessions. The average expression levels increased from ‘Teinturier 2x’ to ‘Teinturier 3x’ to ‘Teinturier 5x’ (Figure 3C,D). These results supported that the spatiotemporal-independent expression level of *VvmybA1* depends on the number of the repetitive 408 bp segments in the promoter and controls, via *VvUFGT*, the anthocyanin content in the leaves. 

### 3.4. Ratio of 3′- and 3′,5′-Substituted Anthocyanins in Leaves Differ Among ‘Teinturier’ Clones

**Figure 4:**
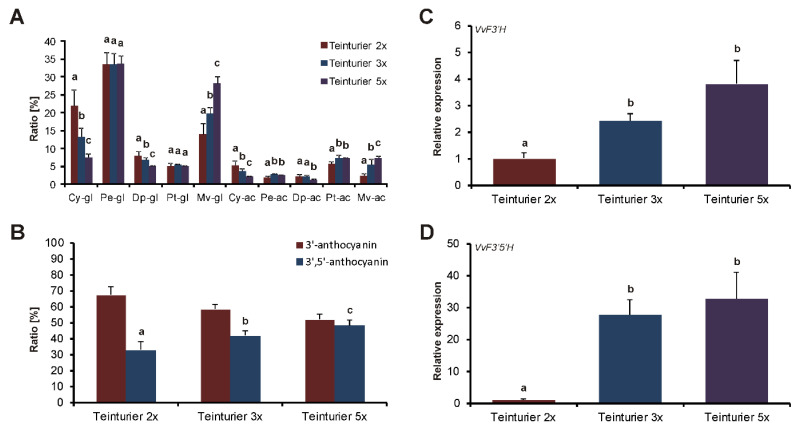
Anthocyanin profiles and ratio of 3′-anthocyanins and 3′,5′-anthocyanins in relation to the expression of *VvF3′H* and *VvF3′5′H* in leaf samples of greenhouse grown ‘Teinturier’ clones with different GCE numbers. (**A**) Anthocyanin profiles. Cy-gl = cyanidin-3-O-glucoside; Pe-gl = peonidin-3-O-glucoside; Dp-gl = delphinidin-3-O-glucoside; Pt-gl = petunidin-3-O-glucoside; Mv-gl = malvidin-3-O-glucoside; ac = acylated anthocyanins. (**B**) Percentage distribution of total anthocyanins classified in 3’-OH-anthocyanins and 3’,5’-OH-anthocyanins. Data represent mean values of six independent replicates; error bars represent the standard deviations (**C**,**D**) Relative expression of *VvF3′H* and *VvF3′5′H* normalized to the expression of ‘Teinturier 2x’. Data represent mean values of six independent replicates; error bars represent standard errors. Different letters indicate statistically significant differences with a *p*-value < 0.05 (Student’s *t*-test, Tukey’s HSD test).

Previous studies indicated that the composition of 3′-hydroxylated and 3′,5′-hydroxylated anthocyanins differ based on the type of tissue (berry skin or flesh) or the variety [17,24]. Therefore, the anthocyanin compositions in leaf samples of the three types of ‘Teinturier’ clones were analyzed and the relative amounts of 3′-hydroxylated and 3′,5′-hydroxylated anthocyanins determined. With increasing GCE number of the *MybA1t* allele, the ratio of these two main anthocyanin groups clearly shifted towards the 3′,5′-hydroxylated anthocyanins from approximately 70:30 in ‘Teinturier 2x’ to 60:40 in ‘Teinturier 3x’ to nearly 50:50 in ‘Teinturier 5x’ (Figure 4A,B; the detailed anthocyanin profiles are given in Table S5). The expression levels of *VvF3′H* and *VvF3′5′H* were determined in parallel since these two genes are mainly responsible for the synthesis of 3′-hydroxylated and 3′,5′-hydroxylated anthocyanins, respectively. Both genes were gradually upregulated with ascending GCE numbers, but the increased relative expression of *VvF3′5′H* from ‘Teinturier 2x’ to ‘Teinturier 5x’ exceeds that of *VvF3′H* clearly from a factor of about 2.7 for *VvF3′H* and 32 for *VvF3′5′H* (Figure 4C,D). The relative higher upregulation of *VvF3′5′H* compared to *VvF3′H* is in agreement to the relative higher 3′,5′-hydroxylated anthocyanins in ‘Teinturier 5x’.

### 3.5. The Influence of the GCE Number on Berry Anthocyanins of the Progeny of ‘Teinturier’

**Figure 5:**
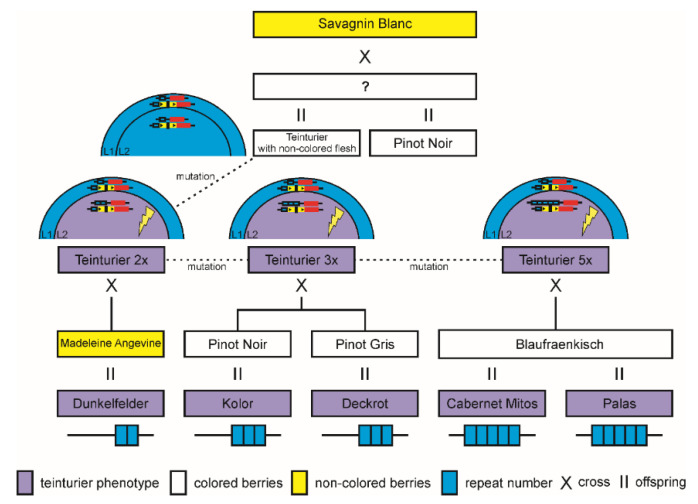
Reconstructed pedigree tree of the variety ‘Teinturier’ and the progeny analyzed in this study.

Fruit anthocyanin content is much more important in grape growing and winemaking than leaf anthocyanin content. ‘Teinturier’ descendants growing in the field and bearing fruit were phenotypically and genetically checked to be true teinturier grapevines with red-fleshed berries. To be genetically as close as possible to the original ‘Teinturier’, all varieties chosen were direct offspring of ‘Teinturier’ (F1 generation). They all were checked to carry, like the parent ‘Teinturier’, one *MybA1t* allele and additionally a non-functional *MybA1a* allele to avoid possible side effects of a second functional *MybA1c* allele. The following two varieties per *MybA1t* allele type were chosen: (i) *MybA1t2*: ‘Teinturier 2x’ and ‘Dunkelfelder’; (ii) *MybA1t3*: ‘Kolor’ and ‘Deckrot’; (iii) *MybA1t5*: ‘Cabernet Mitos’ and ‘Palas’. The full pedigree tree of these varieties including the putative kinship of the ancestor ‘Teinturier’ is displayed in Figure 5.

**Figure 6:**
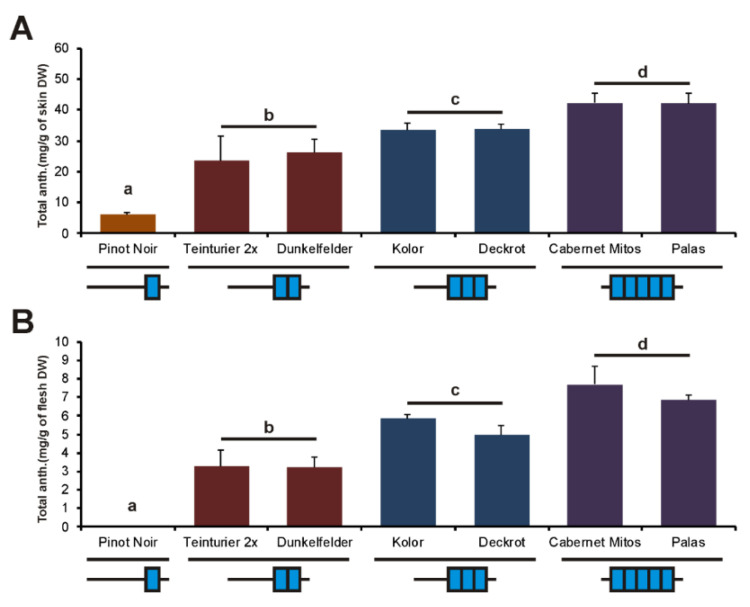
Total anthocyanin content in the skin and flesh of fully ripe berries of ‘Teinturier’ (named ‘Teinturier 2x’) and descendants with different GCE numbers. ‘Pinot Noir’ as reference. (**A**) Total anthocyanin content in the berry skins. (**B**) Total anthocyanin content in the berry flesh. Data represent mean values of three independent replicates; error bars represent the standard deviation. Different letters indicate statistically significant differences with a *p*-value < 0.05 (Student’s *t*-test, Tukey’s HSD test). The repeat number is indicated by blue boxes.

In each variety, the total anthocyanin content of the skin fraction from mature berries was about five to seven times higher than in the berry flesh. Like anthocyanin in the leaves of the ‘Teinturier’ clones, we observed that both berry skin and flesh anthocyanin content of ‘Teinturier’ offspring increased as the GCE number of *MybA1t* increased (Figure 6). ‘Cabernet Mitos’ and ‘Palas’ carry the *MybA1t5* allele and showed the highest anthocyanin concentrations in both tissues. ‘Deckrot’ and ‘Kolor’ with the *MybA1t3* allele had also significantly higher amounts of anthocyanins compared to ‘Teinturier 2x’ and ‘Dunkelfelder’ with the *MybA1t2* allele or the non-teinturier cultivar ‘Pinot Noir’ but reached not the level of the accessions with the *MybA1t5* alleles.

**Figure 7:**
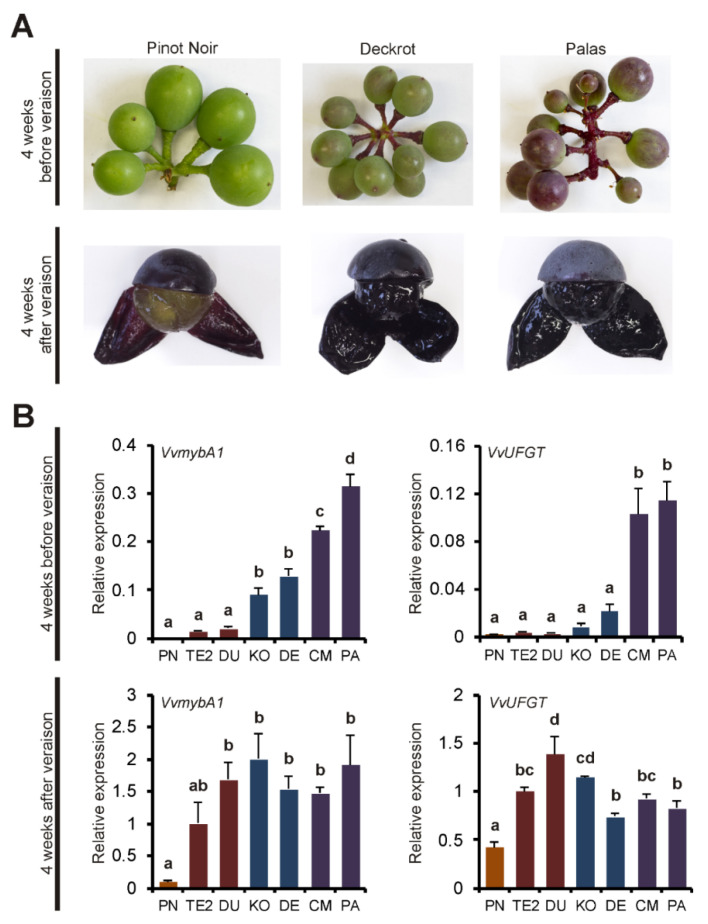
Relative gene expression of *VvmybA1* and *VvUFGT* four weeks before and after veraison of ‘Teinturier’ (named ‘Teinturier 2x’) and descendants with different GCE number. ‘Pinot Noir’ as reference. (**A**) Sample images from berries of the varieties ‘Pinot Noir’ (*MybA1c*), ‘Deckrot’ (*MybA1t3*) and ‘Palas’ (*MybA1t5*). (**B**) Relative expression of *VvmybA1* and *VvUFGT* four weeks before and after veraison normalized to the expression of ‘Teinturier 2x’ four weeks after véraison. Data represent mean values of three independent replicates; error bars represent standard errors. Different letters indicate statistically significant differences with a *p*-value < 0.05 (Student’s *t*-test, Tukey’s HSD test). PN = ‘Pinot Noir’; TE2 = ‘Teinturier 2x’; DU = ‘Dunkelfelder’; KO = ‘Kolor’; DE = ‘Deckrot’; CM = ‘Cabernet Mitos’; PA = ‘Palas’.

Whereas wild-type black-fruited grapevines typically start coloration of the berries with the onset of ripening (veraison), teinturier grapes often begin anthocyanin accumulation before veraison in the pea-sized stage (Figure 7A). At this early developmental stage, transcript analysis of the three different *MybA1t* alleles in the berries showed obvious differences that were proportional to the GCE number of the individual promoters (Figure 7B). For *VvUFGT*, a very similar expression pattern was recorded that lagged slightly behind. In contrast, the relative expression of *VvmybA1* and *VvUFGT* was similar for all teinturiers compared to ‘Pinot Noir’ four weeks after veraison when anthocyanin concentration almost reached its maximum.

## 4. Discussion

### 4.1. The Mutation of ‘Teinturier’ and Its Origin

Berry color is one of the most important fruit traits in viticulture because it is the functional basis for the production of red wines. Almost all grapevine varieties can be assigned to one of two groups (colored and uncolored), depending on whether anthocyanins are present in the berry skin during ripening or not. As berry color mutations easily can be recognized by visual inspection, many sports or clones with berry colors ranging from green/yellow to blue/black have been selected since the rise of viticulture, increasing today’s variety spectrum. The molecular origin for many mutants has been elucidated and is associated with the berry color locus on chromosome 2 [30,59]. The grapevine teinturier phenotype is different from all the previously studied color mutants, since in this case the spatiotemporal specificity of anthocyanin accumulation is altered. As pointed out in Figure 2 and Figure 5, a repetitive structure in the promoter of the *VvmybA1* transcription factor gene leads to the red berry flesh and foliage phenotype in red-fleshed varieties descended from ‘Teinturier’. In previous research, the 408 bp indel was associated with the teinturier phenotype, but the repeating character of this sequence motif was not identified [60]; this is a central and defining feature of the GCE element. The PCR and subsequent sequence analysis of the *VvmybA1* gene of a larger set of ‘Teinturier’ grapevine accessions revealed a 408 bp repeated element (GCE) in the promoter region with variations of two, three or five copies (*Myba1t2*, *MybA1t3* and *MybA1t5*) in the vicinity of the start codon. With the exception of the GCE duplication, the coding sequence itself and the adjacent up and downstream sequences (808 bp 5′ and 404 bp 3′, Figure S1) were identical to the ‘Pinot Noir’ sequences of those regions.

Although previous studies analyzed the properties of teinturier varieties, the specific mutation has not been described until now [24,56,61]. Those studies did not focus on the molecular origin of the teinturier phenotype or used PCR primers located downstream of the GCE element and therefore could not amplify the repetitive structure [25,62]. It also turned out that the PCR amplification of large fragments including the GCE and *MybA1t* gene is very difficult in genotypes with functional (red) *MybA1c* and (red) *MybA1t* haplophases due to identical and competitive primer binding sites and the discrimination towards shorter PCR products. However, after focusing the PCR analysis on L2-specific root tissue (non-functional (white) *MybA1a* and functional (red) *MybA1t*) and resolving the experimental difficulties we identified the repetitive GCE as clearly associated with the red berry pulp and foliage of the original variety ‘Teinturier’ as well as in ‘Teinturier’ offspring. The differences in the coloration of leaves and young berries (prior to veraison) can already visually be observed in teinturiers (Figure 3A and Figure 7A). There is no molecular relationship with other red-fleshed varieties like ‘Gamay Teinturier de Bouze’ or ‘Pinot Teinturier’ that are genetically different and do not carry repetitive GCE sequences (Table 1). This indicates that other red-fleshed mutants have been selected which are based on probably different genetic mechanisms than the GCE. Blast analysis revealed the presence of a single GCE also in the promoter of *VvmybA2* and *VvmybA3* at an almost analogous site of 382 and 380 bp upstream of the start codon, respectively, but with some minor sequence deviations. Related GCE were not found elsewhere in the grapevine PN40024 genome, indicating that this element is a unique sequence of the *VvmybA* genes of the color locus on chromosome 2.

Most repetitive sequences can be classified as orientated short tandem repeats such as SSRs (simple sequence repeats) or longer transposable elements interspersed in the genome [63]. In general, it is assumed that duplications originate in somatic cells from unequal sister chromatid exchange, due to ectopic, allelic or intrachromosomal recombination in the course of double strand break (DSB) repair, replication slippage and transposition [64,65,66]. A recent study demonstrated the formation of tandem duplications as a repair result of induced adjacent single-strand breaks and involvement of short microhomologies at the ends of the insertions [67]. The presence of the 3 bp microhomology TCA at the ends of the GCE (Figure S1) might support the origin of the initial duplication event by the latter model. Further somatic mutations caused by presumable replication slippage led to the allele variants with three and then five perfect GCE copies. Since all three variants are present in ‘Teinturier’ these mutations probably happened successively within this single variety and afterwards were passed to offspring varieties sexually (Figure 5). It cannot be excluded that in addition to the three *MybA1t* alleles identified here, further alleles (e.g., *MybA1t4*, *MybA1t6*, etc.) already exist in not yet analyzed ‘Teinturier’ individuals. It can be expected that allele variants with more than five GCEs would lead to even higher anthocyanin color concentrations than already observed in the *MybA1t5* varieties if there are no negative selective constraints, such as deleterious effects of very high anthocyanin content in leaves. 

### 4.2. The Number of GCE Repeats in the Promoter of the MybA1t Alleles Affects the Spatiotemporal Anthocyanin Formation through Altered Regulation of MybA1-Dependent Genes

The major genes involved in the regulation and biosynthesis of anthocyanins are known. Therefore, teinturiers provide the opportunity to analyze the gene expression in leaf and berry tissues of genotypes with ascending numbers of GCEs affecting the anthocyanin biosynthesis on a quantitative level. In leaves and berries in the stage before veraison, the expression of *VvmybA1* clearly increased and correlated with the number of the repetitive elements in the promoter (Figure 4 and Figure 7). A very similar expression profile was also observed for *VvUFGT* and for *VvF3′H* and *VvF3′5′H*, genes directly controlled by *VvmybA1* [10,21] (Figure 4). The increased expression of *VvF3′H* and *VvF3′5′H* led to a higher amount of 3′-hydroxylated and 3′, 5′-hydroxylated anthocyanins as described by Mu et al. [9]. However, with ascending numbers of GCEs, a shift was observed towards more 3′,5′-hydroxylated than 3′-hydroxylated anthocyanins that are darker in color and further enhances the stronger phenotype of the ‘Teinturier’ accessions with *MybA1t3* and *MybA1t5* alleles (Figure 3). It can therefore concluded that the number of GCEs in the promoter of *VvmybA1* positively enhances the expression of metabolic and regulatory key genes of the anthocyanin biosynthetic pathway and finally the anthocyanin content itself in foliage and fruits. 

**Figure 8:**
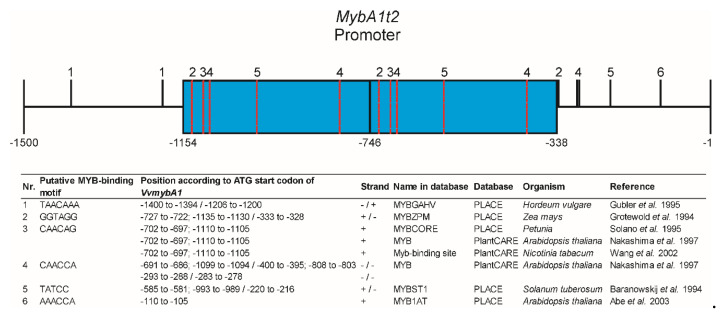
Putative MYB binding motifs within the GCE element and adjacent promoter regions according to the databases PlantCARE [51] and PLACE [52] shown exemplarily for the *MybA1t2* allele. Blue boxes = 2x 408 bp repeats.

In many plant color mutants, elevated anthocyanin production is shown to be linked to increased expressions of R2R3 MYB transcription factors affected by different types of genetic mutations, for example, in apple [68,69], blood orange [70], poplar [71], peanut [72] or cauliflower [73]. In apple, the red-fleshed phenotype was shown to be controlled by the *Mdmyb10* gene with repetitive elements in its upstream regulatory region [74]. The genetic mechanism is explained by autoregulation where the MYB10 protein interacts with a repeated 23 bp sequence in the promoter region of its own gene and is giving rise to ectopic anthocyanin accumulation by enhancing the transcriptional activity. Furthermore, the number of repeated units correlated with the increasing levels of transactivation. To check whether this positive autoregulation is a more general mechanism and the teinturier phenotype is genetically comparable to the red-fleshed apple mutation, the repeated sequence and the adjacent promoter regions of the *MybA1t* alleles were screened for putative MYB-binding motifs. According to the PlantCARE [51] and PLACE [52] databases, the GCE harbors four putative MYB-binding motifs with one binding site each (Figure 8). The motifs were reported for maize *Zea mays* (MYBPZM, [75]), *Petunia* (MYBCORE, [76]), *Solanum tuberosum* (MYBST1, [77]) and *Arabidopsis thaliana* (MYB, myb-binding-site, [78]). Although *VvmybA1t* and *Mdmyb10* show no sequence homology and their repeat units and respective size are different, it is quite conceivable that a regulatory mechanism similar to that described from red-fleshed apples drives the teinturier phenotype in grape. One of these MYB-binding motifs in the GCE alone, a combination of all four, or an interaction with the MYB-binding sites of the adjacent promoter regions could lead to an enhanced *VvmybA1* gene expression by providing additional *cis*-binding sites, causing a stronger positive autoregulation effect and finally higher anthocyanin contents in vegetative tissues. This model would also explain the quantitatively increased anthocyanin formation in relation to the GCE numbers in young leaves and berries. This suggestion is in accordance to recent results from Xie et al. [79] who assumed that the berry flesh color of the teinturier variety Yan 73 is probably positively regulated by VvmybA1 transcriptional activator. In the developmental phase before veraison, the berries of common grapevine varieties like ‘Pinot Noir’ are usually green. In contrast, prior the onset of ripening teinturier grapes showed an early coloring and *VvmybA1* and *VvUFGT* gene expression (Figure 7). An enhanced positive autoregulation of the *MybA1t* alleles might lead to a leaky downregulation of the gene at this stage and subsequently to a temporal earlier induction of anthocyanin formation via an activation of *VvUFGT*, *VvF3’H* and *VvF3’5’H*.

### 4.3. Somatic Mutations and Periclinal Chimeras Are Important Drivers for Increasing Grapevine Diversity

Due to the centuries-long vegetative propagation of old cultivars in viticulture, the level of somatic variation within varieties descending from a single seedling is high [30]. Somatic mutations can either influence the whole plant or affect just one cell layer giving rise to periclinal chimeras that can differ in important traits [80]. For example, ‘Pinot Meunier’, a periclinal chimera of ‘Pinot Noir’, shows an increased hairiness of the shoots [58], whereas ‘Pinot Gris’, another periclinal chimera of ‘Pinot Noir’, lacks anthocyanin biosynthesis in L2 and is therefore unable to produce enough anthocyanins to color red wine [33]. The same mutation can occur multiple times with genetically distinct but phenotypically identical clones as reported for the ‘Pinot’ family [19]. In our work, we identified ‘Teinturier’ as a periclinal chimera with two genetically defined cell layers. The outer cell layer L1 consists of a non-functional *MybA1a* and a functional *MybA1c* allele and should display the original genetic constitution regarding berry color. The inner cell layer L2 has a non-functional *MybA1a* allele and one of the three *MybA1t* alleles, respectively. Since the complete sequence of *MybA1t* from the L2 cell layer, with the exception of the repeats, was identical to the *MybA1c* allele of the L1 layer, the new teinturier alleles arose most probably through mutation of the original *MybA1c* allele of the L2 layer resulting in a periclinal chimera. As gametes arise from the L2 lineage in dicots [81], the variety ‘Teinturier’ passed on the red-fleshed phenotype to its offspring. However, cellular rearrangements in the chimera could lead to homogenization of both cell layers. Owing to the lower level of organization of cell division in the L2 layer, it is thought that the invasion of L2 cells into the L1 cell layer, called “displacement,” is relatively more common [35]. In the case of ‘Teinturier’ the result is the red-flesh genotype in both cell layers. The opposite phenomenon, called “replacement,” is expected to be more rare; this would result in a reversion to the original non-teinturier phenotype. A mutation in ‘Teinturier’ or another red-fleshed variety resulting in a sport with non-colored berry flesh would be of great scientific interest. In contrast to ‘Pinot Gris’, where a loss-of-function mutation in L2 results in a colorless cell layer, ‘Teinturier’ clones show a strong coloration in the berry flesh that is part of L2. We conclude that a gain-of-function mutation in the L2 cell layer of ‘Teinturier’ results in the unique phenotype. 

Before ‘Teinturier’ mutated to the red-fleshed phenotype, the variety should have been a typical red wine cultivar with a functional *MybA1c* allele. ‘Teinturier’ alleles show no additional differences when compared to the ‘Pinot Noir’ *MybA1c* allele (Figure S1) and the SSR loci in the proximity of the color locus are identical between the two varieties (Table S3)—these varieties are very closely related. Previous studies concluded that both ‘Pinot Noir’ and ‘Teinturier’ are in a putative parent-offspring relationship with ‘Savagnin Blanc’ [54,82]. A likelihood ratio test for two a priori relationships (full-siblings against half-siblings) with the software ML-Relate elucidated the pedigree tree of ‘Teinturier’. With a *p*-value of 0.0096, we could conclude that ‘Teinturier’ and ‘Pinot Noir’ are most likely full-siblings sharing the parents ‘Savagnin Blanc’ and an unknown variety with black berries that is the source of the functional color allele.

## 5. Conclusions

We characterized a genetic mutation and its association with the typical phenotype of red berry flesh and foliage of the well-known teinturier grapevine varieties. A 408 bp insertion in the promoter of the *VvmybA1* gene leads to a higher expression of the VvmybA1 gene itself, downstream regulated genes of the anthocyanin biosynthetic pathway and finally the anthocyanin content. Variations of this GCE mutation with two, three and five repeats were found in different ‘Teinturier’ accessions and derived teinturier varieties, which lead to copy number-dependent stronger effects on gene expression and ectopic anthocyanin formation. At the same time, we showed that cultivar ‘Teinturier’” is a periclinal chimera; the original somatic mutation occurred in the L2 layer.

The results highlight the importance of somatic mutations for the vegetatively propagated and perennial grapevine. Grapevine shares molecular mechanisms with some red-fleshed mutants of other fruit crops. Somatic mutations increase genetic diversity and can affect important agronomic traits. The case of ‘Teinturier’ shows that in addition to mutations in the coding sequence of a gene, altered promoters can drive even greater phenotypic diversity, especially if the number of presumable *cis*-acting binding sites for transcription factors is modified. In addition to the mutation and associated phenotype of ‘Teinturier’, we described an example of how variability can further evolve at the mutation site due to putative recombination mechanisms based on microhomology. The three ‘Teinturier’ clones with increasing GCE number represent near-isogenic lines that are ideal for further studies to shed light on the quantitative control of anthocyanin biosynthesis. 

## Figures and Tables

**Table 1:** Grapevine accessions and their *MybA1* allele status (L1 + L2 layer) at the berry color locus. Descendants of ‘Teinturier’ ordered by increasing repeat number of the *MybA1t* allele.

Prime Name (Official Variety Name)	Accession Name (Name in Repository)	Accession No./Origin	MybA1a White	MybA1c Red	MybA1t Repeat Number
Pinot Noir	Spaetburgunder M242	DEU098-1980-006	x	x	-
Pinot Gris	Pinot Gris Cl. Dunkelgrau	DEU098-1980-058	x	x	-
Pinot Teinturier	Pinot Teinturier	DEU454-L-28-4-2	x	x	-
Gamay Teinturier de Bouze	Gamay Teinturier de Bouze	DEU098-1980-196	x	x	-
Teinturier	Teinturier	DEU098-1993-213	x	x	2
Teinturier	Farbtraube	DEU098-2002-003	x	x	2
Teinturier	Farbtraube	DEU098-2011-063	x	x	2
Teinturier	Farbtraube	DEU363-282	x	x	2
Teinturier	Färbertraube	DEU454-L-9-4-2	x	x	2
Teinturier	Teinturier mâle	DEU456-1068	x	x	2
Teinturier	Färbertraube	DEU616	x	x	2
Teinturier	Farbtraube (Fröhlich)	DEU616	x	x	2
Teinturier	Plant rouge femelle	FRA139-303Mtp2	x	x	2
Teinturier	Serzial	FRA139-303Mtp6	x	x	2
Teinturier	Teinturier	DEU616	x	x	3
Teinturier	Plant rouge mâle	FRA139-303Mtp1	x	x	3
Teinturier	Teinturier du Cher PVM	FRA139-0Mtp1749	x	x	3
Teinturier	Rubintraube	DEU456-880	x	x	5
Teinturier	Teinturier du Cher	FRA139-303Mtp7	x	x	5
Dunkelfelder	Dunkelfelder	DEU098-1980-014	x	-	2
Rubired	Rubired	DEU098-1991-123	x	-	2
Titan	Titan	DEU098-1980-459	x	-	2
Teinturier Luebeck	Teinturier male	DEU098-1995-052	x	-	2
Seibel 5437	Seibel 5437	DEU098-2003-093	x	-	2
Golubok	Golubok	DEU098-1988-077	x	-	2
Farbfraenkisch	Farbfraenkisch	DEU454-L-27-5-2	x	-	2
Alicante Henri Bouschet	Alicante Henri Bouschet	DEU098-1990-007	-	x	2
Bouschet Petit	Bouschet Petit	DEU098-2001-016	-	x	2
Karmin	Karmin	DEU098-1991-143	-	x	2
Biborkadarka	Biborkadarka	DEU098-1991-136	-	x	2
Kurucver	Kurucver	DEU098-1991-149	-	x	2
Royalty	Royalty	DEU098-1991-119	-	x	2
Grand Noir	Grand Noir	DEU098-1980-125	-	x	2
Karmrahyut	Karmrahyut	DEU098-1988-079	-	x	2
Morrastel Bouschet	Morrastel Bouschet	DEU098-1980-268	-	x	2
Kolor	Kolor	DEU098-1980-033	x	-	3
Deckrot	Deckrot	DEU098-1980-010	x	-	3
Freiburg 54–64	Freiburg 54–64	DEU098-1980-671	x	-	3
Teinturier femelle (n. i. ^1^)	Teinturier femelle	DEU456-1127	x	-	3
Accent	Accent	DEU098-2013-031	x	-	3
Dakapo	Dakapo	DEU098-2011-046	-	x	3
Cabernet Mitos	Cabernet Mitos	DEU098-2010-046	x	-	5
Palas	Palas	DEU098-2010-049	x	-	5

^1^ n. i. = non identified.

## Supplementary Materials

The following are available online at https://www.mdpi.com/2073-4425/11/8/891/s1. Figure S1: Alignment of the *MybA1t2* allele of ‘Teinturier’ and the *MybA1c* allele of ‘Pinot Noir’. Figure S2: *Eco*RI restriction analysis of the *VvmybA1* amplicons. Table S1: Analyzed grapevine accessions and their origin. Table S2: Information of primers used for berry color specific SSR-marker analysis. Table S3: Genotypic data of 15 SSR loci from the analyzed grapevine accessions. Table S4: List of Likelihoods for the kinship of ‘Savagnin Blanc’, ‘Pinot Noir’ and ‘Teinturier’. Table S5: Anthocyanin composition (%) in leaf samples of ‘Teinturier’ clones with different GCE number.

## Data Deposition

The PCR sequence data have been deposited in the GenBank database (http://www.ncbi.nlm.nih.gov/genbank) with the accession numbers MT482339 *(VvmybA1t2*), MT482340 (*VvmybA1t3*) and MT482341 (*VvmybA1t5*).
