# Supplementary material for: Color Intensity of the Red-Fleshed Berry Phenotype of Vitis vinifera Teinturier Grapes Varies Due to a 408 bp Duplication in the Promoter of VvmybA1
Source: Genes (Basel). 2020 Aug 5;11(8):891. doi: 10.3390/genes11080891 (PMC7464560; doi:10.3390/genes11080891)
Supplement: Supplementary file 1 [file genes-11-00891-s001.pdf]

genes

Supplements

Color intensity of the red-fleshed berry phenotype of *Vitis vinifera* teinturier grapes varies due to a 408 bp duplication in the promoter of *VvmybA1*

Franco Röckel[1], Carina Moock[1], Ulrike Braun[1], Florian Schwander[1], Peter Cousins[2], Erika Maul[1], Reinhard Töpfer[1], Ludger Hausmann[1],*

[1] Institute for Grapevine Breeding Geilweilerhof, Julius Kühn-Institut, Federal Research Centre for Cultivated Plants, 76833 Siebeldingen, Germany; franco.roeckel@julius-kuehn.de (F.R.); carina.moock@julius-kuehn.de (C.M.); ulrike.braun@julius-kuehn.de (U.B.); florian.schwander@julius-kuehn.de (F.S.); erika.maul@julius-kuehn.de (E.M.); reinhard.toepfer@julius-kuehn.de (R.T.)

[2] E. & J. Gallo Winery, Modesto, California 95354, United States of America; peter.cousins@ejgallo.com (P.C.)

* Correspondence: ludger.hausmann@julius-kuehn.de; Tel.: +49-6345-41-117

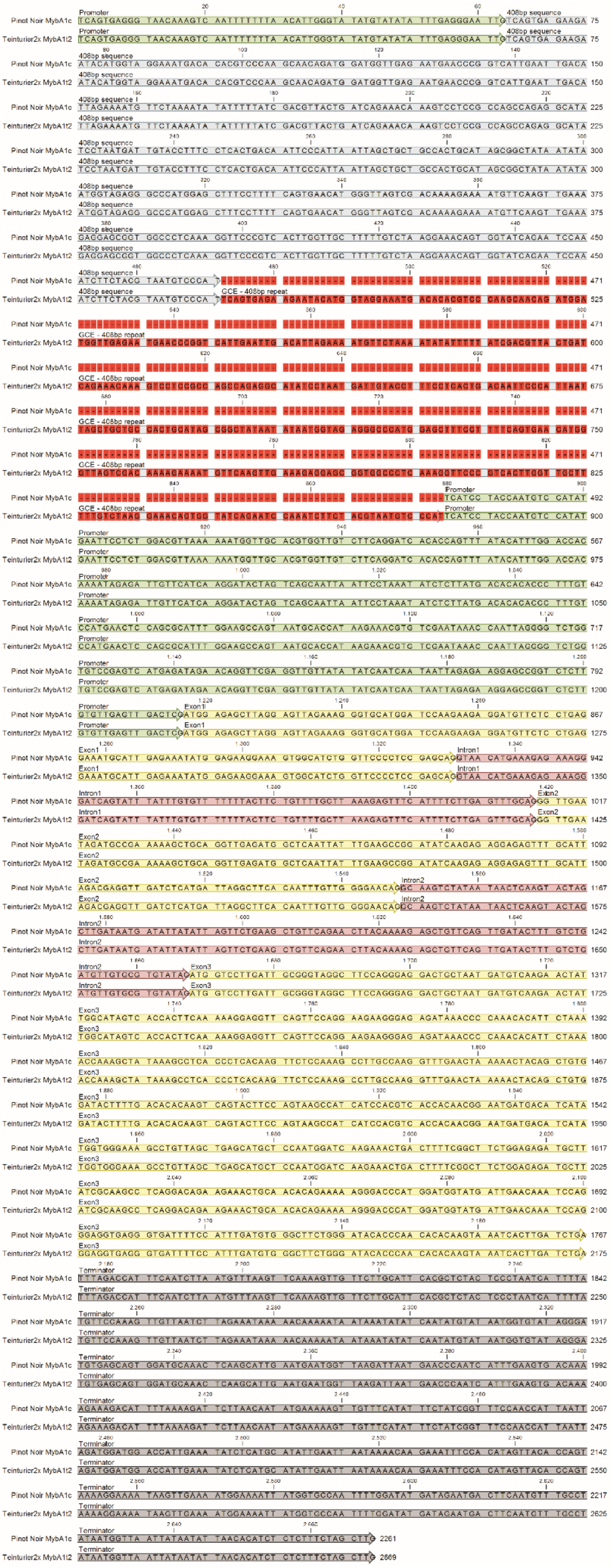

Figure S1. Alignment of the *MybA1t2* allele of 'Teinturier' and the *MybA1c* allele of 'Pinot Noir'. Differences are indicated in red.

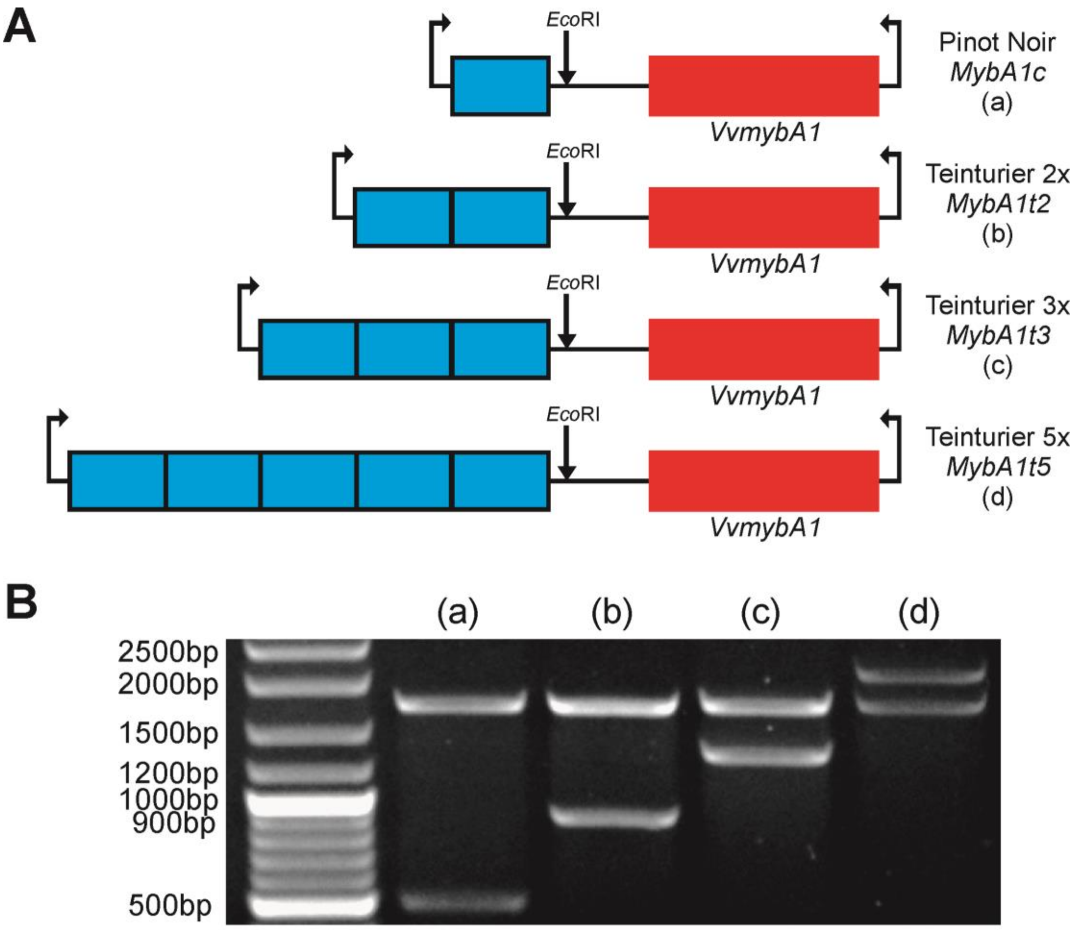

C

Allele	Length [bp]	Fragment 1 [bp]	Fragment 2 [bp]	Repeat number
MybA1c	2261	1768	493	1 (wildtype)
MybA1t2	2669	1768	901	2
MybA1t3	3077	1768	1309	3
MybA1t5	3893	1768	2125	5

Figure S2. *Eco*RI restriction analysis of the *VvmybA1* amplicons. Leaf-DNA sample ('Pinot Noir') and the root-DNA samples ('Teinturier 2x', 'Teinturier 3x' and 'Teinturier 5x') shown in Fig. 1. (A) Schematic overview of the amplicons with the relative position of the *Eco*RI restriction site. (B) Agarose gel of the digested amplicons. (C) Fragment lengths based on amplicon sequencing.

Table S1. Analyzed grapevine accessions and their origin according to the *VIVC* database (www.vivc.de). Tissue types used for anthocyanin analysis are indicated.

Prime name	Variety number *VIVC*	Accession name	Accession number	Origin	Anthocyanin measurements	Referred in this study as
Pinot Noir	9279	Spaetburgunder M242	DEU098-1980-006	JKI Siebeldingen	berry skin, berry flesh	
Pinot Gris	9275	Pinot Gris Cl. Dunkelgrau	DEU098-1980-058	JKI Siebeldingen	leaf	
Pinot Teinturier	9283	Pinot Teinturier	DEU454-L-28-4-2	HGU Geisenheim		
Gamay Teinturier de Bouze	4379	Gamay Teinturier de Bouze	DEU098-1980-196	JKI Siebeldingen		
Teinturier	12304	Teinturier	DEU098-1993-213	JKI Siebeldingen	leaf, berry skin, berry flesh	Teinturier 2x
Teinturier	12304	Farbtraube	DEU098-2002-003	JKI Siebeldingen		
Teinturier	12304	Farbtraube	DEU098-2011-063	JKI Siebeldingen		
Teinturier	12304	Farbtraube	DEU363-282	DLR Neustadt		
Teinturier	12304	Färbertraube	DEU454-L-9-4-2	HGU Geisenheim		
Teinturier	12304	Teinturier mâle	DEU456-1068	LVWO Weinsberg		
Teinturier	12304	Färbertraube	-	BSA Hassloch		
Teinturier	12304	Farbtraube (Fröhlich)	-	BSA Hassloch		
Teinturier	12304	Plant rouge femelle	FRA139-303Mtp2	INRA Vassal		
Teinturier	12304	Serzial	FRA139-303Mtp6	INRA Vassal		
Teinturier	12304	Teinturier	-	BSA Hassloch	leaf	Teinturier 3x
Teinturier	12304	Plant rouge mâle	FRA139-303Mtp1	INRA Vassal		
Teinturier	12304	Teinturier du Cher PVM	FRA139-0Mtp1749	INRA Vassal		
Teinturier	12304	Rubintraube	DEU456-880	LVWO Weinsberg	leaf	Teinturier 5x
Teinturier	12304	Teinturier du Cher	FRA139-303Mtp7	INRA Vassal		
Dunkelfelder	3724	Dunkelfelder	DEU098-1980-014	JKI Siebeldingen	berry skin, berry flesh	
Rubired	10308	Rubired	DEU098-1991-123	JKI Siebeldingen		
Titan	12531	Titan	DEU098-1980-459	JKI Siebeldingen		
Teinturier Luebeck	23366	Teinturier male	DEU098-1995-052	JKI Siebeldingen		
Seibel 5437	11110	Seibel 5437	DEU098-2003-093	JKI Siebeldingen		
Golubok	4888	Golubok	DEU098-1988-077	JKI Siebeldingen		
Farbfraenkisch	4053	Farbfraenkisch	DEU454-L-27-5-2	HGU Geisenheim		
Alicante Henri Bouschet	304	Alicante Henri Bouschet	DEU098-1990-007	JKI Siebeldingen		

Bouschet Petit	1619	Bouschet Petit	DEU098-2001-016	JKI Siebeldingen	
Karmin	5999	Karmin	DEU098-1991-143	JKI Siebeldingen	
Biborkadarka	1339	Biborkadarka	DEU098-1991-136	JKI Siebeldingen	
Kurucver	6573	Kurucver	DEU098-1991-149	JKI Siebeldingen	
Royalty	10288	Royalty	DEU098-1991-119	JKI Siebeldingen	
Grand Noir	5012	Grand Noir	DEU098-1980-125	JKI Siebeldingen	
Karmrahyut	6002	Karmrahyut	DEU098-1988-079	JKI Siebeldingen	
Morrastel Bouschet	8009	Morrastel Bouschet	DEU098-1980-268	JKI Siebeldingen	
Kolor	6388	Kolor	DEU098-1980-033	JKI Siebeldingen	berry skin, berry flesh
Deckrot	3482	Deckrot	DEU098-1980-010	JKI Siebeldingen	berry skin, berry flesh
Freiburg 54- 64	4240	Freiburg 54- 64	DEU098-1980-671	JKI Siebeldingen	
Teinturier femelle (non identified)	24854	Teinturier femelle	DEU456-1127	LVWO Weinsberg	
Accent	20540	Accent	DEU098-2013-031	JKI Siebeldingen	
Dakapo	14728	Dakapo	DEU098-2011-046	JKI Siebeldingen	
Cabernet Mitos	15499	Cabernet Mitos	DEU098-2010-046	JKI Siebeldingen	berry skin, berry flesh
Palas	16871	Palas	DEU098-2010-049	JKI Siebeldingen	berry skin, berry flesh

Table S2. Information of primers used for berry color specific SSR-marker analysis. The physical position refers to the 12x version of the PN40024 reference genome (http://ensembl.gramene.org/Vitis_vinifera/Info/Index).

Marker name	Chromosome	Physical position	Forward primer	Reverse primer	Reference
GF02-55	2	13998165	AAAATTGAAGGACAGGAGGAGG	GCAAGGCTGGTCTACTCAGAAA	Huber *et al.* (2016)
VVNTM1	2	14149178	CCACGCCACTATTGCTAAAC	TGCACCGTATCAAGATCATGTC	Fournier-Level *et al.* (2009)
GF02-61	2	14187240	GCAAACCTCCAGAACCTGATTT	TATTGGTCGAGAAGGAATGTGG	This study
GF02-62	2	14192053	GGGATTTGTTAAGGAGATTTGC	GATGACAGTTCAGAGGTTGGC	This study
GF02-68	2	14252332	CCCCTGCCATCTCCATGC	GGTCGTTGCATCATTTTCTCCA	This study
VVNTM5	2	14325217	AGGAGGAATCCACATCAAAAGA	TGATTCAAAGGAATAAATAACCATCA	Fournier-Level *et al.* (2009)

Table S3. Genotypic data of 15 SSR loci from the analyzed grapevine accessions.

Prime name	VVS2	VVMD5	VVMD7	VVMD25	VVMD27	VVMD28	VVMD32	VrZag62	VrZag79
Pinot Noir	137:151	230:240	239:243	239:249	186:190	218:236	240:272	188:194	239:245
Teinturier (all 15 clones)	137:151	240:240	239:243	239:249	180:190	228:236	272:272	188:194	245:255
Dunkelfelder	135:151	238:240	243:247	249:255	180:195	228:244	240:272	188:204	249:255
Kolor	151:151	240:240	239:243	249:249	180:186	218:236	272:272	188:194	239:255
Deckrot	137:151	230:240	239:243	249:249	190:190	218:236	240:272	188:194	245:245
Cabernet Mitos	137:143	228:240	239:239	239:249	180:195	228:246	250:272	194:194	251:255
Palas	137:143	240:242	239:239	239:249	180:195	228:246	272:272	194:204	251:255

Genome-wide SSR-markers used for genotyping

Prime name	GF02-55	VVNTM1	GF02-61	GF02-62	GF02-68	VVNTM5
Pinot Noir	215:229	163:169	283:286	373:385	374:390	294:308
Teinturier (all 15 clones)	215:229	163:169	283:286	373:385	374:390	294:308
Dunkelfelder	217:229	163:169	283:286	373:385	374:390	294:308
Kolor	215:229	163:169	283:286	373:385	374:390	294:308
Deckrot	215:229	163:169	283:286	373:385	374:390	294:308
Cabernet Mitos	217:229	163:169	283:286	373:385	374:390	294:308
Palas	217:229	163:169	283:286	373:385	374:390	294:308

SSR-markers specific for the berry color locus on chr 2

Table S4. List of Likelihoods for the kinship of 'Savagnin Blanc', 'Pinot Noir' and 'Teinturier'. Calculated with ML Relate (Kalinowski *et al.*, 2006) based on the marker dataset of Lacombe *et al.* (2013). Output Ln(Likelihood) for each of the four following relationships: U = unrelated; HS = half sibs; FS = full sibs; PO = parent / offspring. R = relationship with the highest likelihood; LnL(R) = log likelihood of R; Delta Ln(L) = log likelihood differences for the other relationships compared to the highest likelihood LnL(R).

				Delta Ln(L)			
**Variety 1**	**Variety 2**	**R**	**LnL(R)**	**U**	**HS**	**FS**	**PO**
Savagnin Blanc	Teinturier	PO	-92.88	14.34	5.40	6.31	-
Savagnin Blanc	Pinot Noir	PO	-100.17	13.42	5.16	4.07	-
Teinturier	Pinot Noir	FS	-97.72	9.79	2.66	-	Not possible

Table S5. Anthocyanin composition [%] in leaf samples of 'Teinturier' clones with different GCE number. Cy-gl = cyanidin-3-O-glucoside; Pe-gl = peonidin-3-O-glucoside; Dp-gl = delphinidin-3-O-glucoside; Pt-gl = petunidin-3-O-glucoside; Mv-gl = malvidin-3-O-glucoside; ayl = acetylated 3-O-glucoside anthocyanins; cum = coumarylated 3-O-glucoside anthocyanins.

Clone	Cy-gl	Pe-gl	Dp-gl	Pt-gl	Mv-gl	Cy-ayl	Pe-ayl	Dp-ayl	Pt-ayl	Mv-ayl	Cy-cum	Pe-cum	Dp-cum	Pt-cum	Mv-cum
Teinturier 2x	21.87±4.54	33.45±3.29	8.01±1.25	5.11±0.8	13.85±3.18	-	0.75±0.39	-	-	-	5.33±1.19	5.81±0.65	2.13±0.63	1.27±0.13	2.43±0.66
Teinturier 3x	13.28±2.36	33.54±3.05	6.79±0.75	5.49±0.25	19.62±1.86	-	1.04±0.14	0.36±0.11	0.38±0.07	0.77±0.18	3.68±0.73	6.81±0.97	1.74±0.31	1.67±0.29	4.83±1.27
Teinturier 5x	7.53±1.08	33.79±2.15	5.02±0.32	4.94±0.37	28.2±1.96	-	1.2±0.1	0.27±0.03	0.22±0.02	1.18±0.09	2.21±0.19	7.01±0.17	0.98±0.12	1.33±0.13	6.11±0.66
